# Parenting and Emotional and Behavioral Difficulties in a General Population Sample of Adolescents: The Mediating Role of Emotional Dysregulation

**DOI:** 10.3390/children11040435

**Published:** 2024-04-05

**Authors:** Martina Smorti, Annarita Milone, Luisa Fanciullacci, Alessia Ciaravolo, Carmen Berrocal

**Affiliations:** 1 Department of Surgical, Medical and Molecular Pathology, and Critical Care Medicine, University of Pisa, 56126 Pisa, Italy; martina.smorti@unipi.it (M.S.); l.fanciullacci@studenti.unipi.it (L.F.); a.ciaravolo@studenti.unipi.it (A.C.); carmen.berrocalmontiel@unipi.it (C.B.); 2 IRCCS Fondazione Stella Maris, Scientific Institute of Child Neurology and Psychiatry, 56128 Calambrone, Italy; 3 International Lab of Clinical Measurements, University of Florence, Viale Pieraccini, 6, 50139 Florence, Italy

**Keywords:** parenting, internalizing problems, externalizing problems, adolescence, emotional dysregulation

## Abstract

Research has shown that both parenting and emotional dysregulation are associated with mental health outcomes in youth. This cross-sectional research was developed to replicate these noted findings and explore the mediating role of emotional dysregulation to explain the relationship between parenting and emotional and behavioral difficulties (internalizing and externalizing problems) in adolescents. A total of 104 adolescents (61.5% females; M = 15.62 yrs., SD = 1.38) participated in the study. Participants completed the Parental Bonding Instrument (measuring care, promotion of autonomy, and overprotection) referring to both the mother and father, the Difficulties in Emotion Regulation Scale, and the Youth Self-Report. The results showed that difficulties in emotion regulation fully mediated the relationship between overprotection (in both parents) and low maternal care with internalizing problems, on the one hand, and the relationship between maternal overprotection and low care (in both parents) with externalizing problems, on the other hand. Furthermore, emotional dysregulation partially mediated the effect of paternal care on internalizing problems. These findings help to clarify one of the mechanisms through which parenting can affect mental health in youth. Theoretical and clinical implications are discussed.

## 1. Introduction

Adolescence, the transitional period between childhood and young adulthood, is a natural phase of learning and adjustment [1], in which individuals need to explore their identity to reach a subjective feeling of self-sameness across different times and contexts [2,3]. During this development phase, individuals are called to rebalance their social relationships by achieving a growing independence from their parents, while integrating themselves into the peer group [4]. All these changes can constitute stressors that lead adolescents to experience more intense and frequent negative emotions and higher mood swings [5,6], which in turn may increase the risk of poor mental health outcomes in terms of emotional/behavioral difficulties up to internalizing (i.e., anxiety/depression, somatic complaints, and withdrawn) and externalizing (i.e., delinquent and aggressive behavior) disorders [7,8]. Both an impairment in emotion regulation strategies and parenting have emerged as important predictors of emotional and behavioral difficulties among adolescents.

Emotion regulation is usually defined as an automatic process involved in “monitoring, evaluating, and modifying emotional reactions, especially their intensive and temporal features, to accomplish one’s goals” [9] (pp. 27–28). Emotion regulation can occur in different ways on the cognitive (e.g., attempting to solve the problem or ruminating on the causes of emotions) or behavioral (e.g., attempting to completely distract oneself from the emotion or conversely adopting a withdrawal mode) level [10,11]. When they have to deal with unpleasant feelings and thoughts, individuals with emotional self-regulation abilities tend to use reappraisal, problem-solving, and acceptance, which are considered adaptive strategies since they increase emotional well-being [12]. On the contrary, individuals with a deficit in emotional self-regulation may be prone, when they experience unpleasant emotions, to use more maladaptive strategies such as rumination (that increases the risk for internalizing problems), avoidance, or impulsive and aggressive behavior (increasing the risk for externalizing problems) [13,14,15,16,17].

On the other hand, the family is one of the main daily living contexts of adolescents and, even though further longitudinal and prospective evidence is needed [18], the literature suggests that experienced parenting practices can influence the development of emotional and behavioral difficulties in terms of internalizing and externalizing disorders [19,20,21,22]. Parenting has been defined by three main dimensions as follows: care, overprotection, and promotion of autonomy [23]. Care refers to the parental ability to understand the emotional needs of their offspring [23,24]. It reflects a continuum from supportive, warm, affectionate, and empathetic style (high levels of care) to cold, neglectful, and unresponsive parental style (low levels) [23,24,25,26]. 

Overprotection refers to the measure in which parents tend to make the offspring dependent [23,27]. High levels of overprotection describe excessive levels of protection, considering the developmental level of the child up to parental intrusion. On the other hand, low levels of overprotection refer to the appropriate parental protection expressed by the attitudes of guiding offspring’s behavior toward acceptable standards. Finally, the promotion of autonomy regards the degree of parental support to autonomous choice by offspring [25]. High levels in this factor define the parental encouragement of the offspring’s possibility to decide in autonomy and a respectful attitude toward the child’s independence, while low levels indicate the discouragement of autonomous choice by offspring.

Parenting characterized by high care, high promotion of autonomy, and low overprotection tends to promote higher psychological well-being [28] and lower externalizing and internalizing problems [19,29,30,31,32]. On the other hand, high parental overprotection and low care and autonomy promotion are associated with emotional problems and internalizing disorders during adolescence [26,31,32,33,34,35,36].

Although parenting seems to be an important predictor of mental health outcomes in youth, further research is needed to better understand the processes through which parenting may affect mental health. Several factors have been proposed as mediators between parenting and emotional/behavioral problems such as low self-esteem [37], attentional deficits [38], and difficulties in emotion recognition in terms of alexithymia [36,37,38,39]. For example, previous related work on clinical populations showed that overprotective and careless parenting increases adolescents’ difficulty in identifying and describing emotional experiences due to an elevated level of alexithymia increasing internalizing problems [36,39]. 

Thus, warm parenting characterized by emotional validation of shared emotions by adolescents seems to promote the development of adequate emotional self-regulation from childhood to adulthood [40,41,42], which in turn may foster high psychological and social well-being in offspring [43,44,45]. On the contrary, a cold, neglectful, unresponsive, or invalidating parental style can increase youth’s distress and teach offspring that emotions are unacceptable and cannot be tolerated by their parents, thus limiting the opportunities to learn more effective ways of dealing with unpleasant emotions [46]. Moreover, parenting practices associated with control, intrusion, and over-protection predict emotion dysregulation both in children and adolescents [47,48,49,50,51].

Overall, the previous literature showed that (a) both emotional regulation deficits and parenting predict adolescents’ emotional and behavioral difficulties up to internalizing and externalizing disorders and (b) parenting practices influence the ability to self-regulate emotion. Given the impact of parenting on emotional dysregulation, and the role of the latter on internalizing and externalizing disorders, it is reasonable to hypothesize that emotional dysregulation may act as a mediator between parenting and internalizing/externalizing problems. Previous work on this issue is scarce, focused only on some parenting dimensions, and somewhat inconsistent. One study showed that adolescents’ difficulties in emotion regulation mediated the link between fathers’ psychological control and emotional symptoms in a sample of clinical adolescents with anorexia nervosa [52]. Walton et al. [53] found that adaptive emotional regulation strategies mediated the effect of maternal warmth on behavioral problems in a community sample of adolescents. However, Boullion et al. [54] found that warmth predicted internalizing but not externalizing disorders and that this effect was mediated by emotional regulation in a general population sample of adolescents. 

According to the above considerations, the present study aimed to examine whether emotional dysregulation mediates the relationship between parenting (i.e., care, overprotection, and promotion of autonomy) and internalizing and externalizing problems in a non-clinical sample of adolescents. Consistent with the above past research, it was expected that the contribution of parenting to predicting internalizing/externalizing problems and emotional dysregulation would be statistically significant. Further, in line with the literature [52,53,54], it was hypothesized that emotional dysregulation would mediate the relationship between parenting and internalizing/externalizing problems. It is worth noting that in line with other studies, e.g., [26,36], we aimed to examine adolescents’ perceptions of both their mothers’ and fathers’ parenting, while most research has focused only on one parent’ parenting (mainly maternal, e.g., [32,35,53,55], despite some exceptions [52]), hence neglecting the potential effect of both parents’ parenting.

## 2. Materials and Methods

### 2.1. Participants and Procedure

This study used a cross-sectional design and a convenience sample of adolescents. This study was approved by the Bioethical Committee of the University of Pisa (Italy) (nr. 13/2022). Participants were recruited from students attending high schools in Pisa. Inclusion criteria were (a) age between 14 and 18 years; (b) fluent in the Italian language; and, (c) informed written consent from the participant (and from the parents or guardians if the participant was under the age of 18). 

The adolescents were informed about the objectives and procedures of this study and signed the consent form before participation. Adolescents who agreed to participate in this study completed a questionnaire packet consisting of the measures described below (see Section 2.2). Data were collected anonymously, participation in this study was voluntary, and no incentive was offered to respondents. A total of 104 participants aged 14–19 years (61.5% females, *n* = 64; mean age = 15.62 years; SD = 1.38) were included in this study.

### 2.2. Measures and Instruments

Participants filled out a form to collect socio-demographic data (i.e., sex, age) and the battery of self-report questionnaires described below.

In order to measure participants’ perceptions of their relationship with their parents, the Italian adaptation [56] of the Parental Bonding Instrument (PBI) [57] was used. This self-report questionnaire consists of two parallel versions for measuring self-perceived relationships separately for each parent. Each version consists of 21 items that assess the following three dimensions of parenting: care, promotion of autonomy, and overprotection. Participants were asked to indicate the degree of agreement with the item statement using a 4-point Likert scale (from 0 to 3). Higher scores in the PBI dimensions indicate high levels of care, encouragement toward autonomy, and overprotection. In the present study, Cronbach’s alpha coefficients were 0.92 and 0.89 for the paternal and maternal care dimensions, respectively; 0.88 and 0.81 for the paternal and maternal autonomy factors, respectively; and 0.64 for both the paternal and maternal overprotection domains.

The Italian Adaptation [58] of the Difficulties in Emotion Regulation Scale—Short Form (DERS-SF) [59] was used to measure emotional regulation strategies. The DERS-SF is composed of 20 items assessing difficulties within the following six different dimensions: (a) poor awareness of emotional responses, (b) lower levels of emotional clarity, (c) non-acceptance of emotional responses, (d) limited access to emotion regulation strategies perceived as effective, (e) impulsivity when experiencing unpleasant emotions, and (f) difficulties engaging in goal-directed behaviors when experiencing unpleasant emotions. DERS-SF items are rated on a 5-point scale (from 1 to 5), with higher total scores indicating higher levels of emotional dysregulation. For the purposes of this study, only the DERS-SF total score was used. In this study, Cronbach’s alpha coefficient was 0.84 for the DERS-SF total score.

The Italian version (ASEBA) of the Youth Self Report (YSR/11-18) [60] was used to measure emotional and behavioral problems. The YSR contains 112 items rated on a 3-point scale (from 0 to 2). The YSR consists of five narrow-band scales that are grouped into the following two broadband scales: the internalizing (withdrawn, somatic complaints, and anxious/depressed scales) and the externalizing (delinquent and aggressive behavior scales) scales. For the purpose of this study, only the two broadband scales were used. Alpha coefficients were 0.89 and 0.84 for the Internalizing and the Externalizing scales, respectively, in our sample.

### 2.3. Statistical Analyses

The statistical analyses were performed using Statistical Package for Social Sciences (SPSS) version 20.0. Preliminary analyses included correlations between the main variables of the study as well as Harman’s single-factor test to examine potential common method biases. Hierarchical regression analyses were conducted to test the mediational models. According to Baron and Kenny [61], support for mediational hypotheses is provided if (a) PBI dimensions are significant predictors of internalizing and externalizing problems; (b) PBI dimensions significantly predict the hypothesized mediator (i.e., difficulties in emotion regulation); and (c) difficulties in emotion regulation significantly predict internalizing and externalizing problems, while PBI dimensions do not remain significant predictors of externalizing and internalizing disorders once the mediator is entered into the model.

## 3. Results

### 3.1. Preliminary Analyses

The descriptive statistics and correlation coefficients for the main variables in this study are presented in Table 1. The results from Harman’s test showed that the amount of variance explained by the first factor was 13.92%, which is far less than the recommended cut-off of 50% [62], hence indicating that common method variance did not affect the dataset. 

Correlations between socio-demographic variables (i.e., age and gender) and behavioral and emotional problems were explored to identify potential confounders. Age was significantly and negatively associated with externalizing problems. In addition, gender significantly correlated with both internalizing and externalizing problems. Females showed higher internalizing problems (M = 24.25, SD = 10.36) than males (M = 18.65, SD = 9.36) (t = −2.78, *p* = 0.006), while males showed higher externalizing problems (M = 16.32, SD = 8.46) than females (M = 12.00, SD = 6.26) (t = 2.79, *p* = 0.007). Hence, age and gender were included as covariates in the subsequent regression analyses.

### 3.2. Regression Analyses for Testing the Mediational Models

Table 2 shows the results from regression analyses conducted to explore whether parenting styles significantly predict internalizing and externalizing problems. Age and gender were included as covariates in the first step of each equation, and each PBI dimension was entered as the independent variable. All the final models to predict internalizing and externalizing problems were significant. Except for the promotion of autonomy dimension for both parents, all the PBI factors (i.e., high care and low overprotection) emerged as significant predictors of internalizing problems in the last step of each equation, and they accounted for an additional 4 to 16 percent of the variance in internalizing problems. Regarding the externalizing problems, high maternal overprotection, and low maternal/paternal care emerged as significant predictors in the last step of each equation. These dimensions accounted for an additional 3 to 7 percent of the variance in externalizing symptomatology.

Regarding socio-demographic variables, in the first step of each model gender and age accounted significantly for 5% (for internalizing problems; F = 3.43; *p* < 0.05) to 10% (for externalizing problems; F = 6.87; *p* ≤ 0.01) of the variance in the outcome measures. In the last step of each equation, gender was a significant predictor of internalizing and externalizing problems, with males showing higher levels of externalizing difficulties and females internalizing problems. Age was a significant predictor of only externalizing problems, with younger participants showing higher levels of externalizing difficulties.

Next, regression analyses were conducted to examine whether PBI dimensions significantly predict difficulties in emotion regulation (see Table 3). As before, each PBI dimension was entered as the independent variable, while socio-demographics were included as covariates in the first step. Except for models that included the promotion of autonomy dimension, all final models were statistically significant, with care and overprotection dimensions accounting for an additional 7% to 16% of the variance in emotional dysregulation. Both the care and overprotection dimensions emerged as significant predictors of difficulties in emotion regulation after controlling for the effect of socio-demographic variables.

To test the final step of the mediational models, difficulties in emotion regulation and each PBI dimension were entered together in the second block of each equation (with age and gender included as covariates in the first block). These analyses were conducted only for those PBI dimensions that in prior analyses proved to significantly predict emotional dysregulation as well as internalizing and/or externalizing problems (see Table 4). All final models predicting internalizing and externalizing scores were statistically significant, with difficulties in emotion regulation and parenting dimensions accounting for an additional 41 to 44 percent of the variance in internalizing problems, and an additional 15% of the variance in externalizing difficulties. As expected, difficulties in emotion regulation emerged as a significant predictor of internalizing and externalizing disorders in all equations. Moreover, as hypothesized, the overprotection (in both parents) and maternal care dimensions did not remain as significant predictors of internalizing disorders when DERS scores were included in the models, supporting the mediational model for these parenting styles. The effect of paternal care on internalizing difficulties was reduced when DERS scores were included in the model, hence suggesting a partial mediation to explain the effect of paternal low care on internalizing difficulties. Furthermore, care (in both parents) and maternal overprotection did not predict externalizing problems when DERS scores were included in the models, hence supporting the mediational hypotheses also for these problems.

The results from the Sobel test, conducted to examine the statistical significance of the mediation effect, supported that difficulties in emotion regulation fully mediated the relationship between overprotection (z = 2.56, *p* = 0.01 for maternal scores; z = 2.84 for paternal scores) and low care (z = −3.04, *p* < 0.01 for maternal scores; z = −3.78 for paternal scores) with internalizing problems, on one hand, and the relationship between low care (z = −2.45, *p* = 0.01 for maternal scores; z = −2.66, *p* < 0.01 for paternal scores) and maternal overprotection (z = 2.20, *p* < 0.05) with externalizing problems, on the other hand.

## 4. Discussion

This study aimed to investigate the mediator role of emotion dysregulation to explain the previously demonstrated relationship between parenting and internalizing/externalizing problems. In line with the literature, emotion dysregulation emerged as a significant predictor of internalizing/externalizing problems [13,14,15,16,17]. Difficulties in managing negative emotions may influence both the persistence of sad feelings and subsequent depressive and anxious symptoms (internalizing problems) and the impulsivity that increases the risk for externalizing problems [63]. Overall, these results also provide further support for emotion regulation as a cross-disorder factor predicting quality of life and treatment duration in child psychopathology [64].

Consistent with previous research [26,33,34,35,36], both maternal and paternal high overprotection and low care were significant predictors of internalizing problems. It is worth noting that we explored adolescents’ perceptions of both their mothers’ and fathers’ parenting, while most studies examining parenting have focused only on mothers, e.g., [35,53,55]. Although the choice to focus on maternal parenting may be justified by the fact that fathers, compared with mothers, report less involvement and more punitive and less sensitive reactions to children’s emotions [65], neglecting the father’s role in emotional dysregulation may potentially reinforce implicit messages that mothers are to blame for their offspring’s developmental difficulties [66].

Interestingly, while both maternal and paternal care dimensions were significant predictors for externalizing problems, only maternal overprotection proved to be a significant predictor of externalizing difficulties in this study. The results concerning the effect of low parental care on adolescents’ difficulties are in line with previous findings, e.g., [24,31,34]. Indeed, previous studies suggested that a lack of affection may interfere with offspring’s ability to regulate arousal [24] and may increase adolescents’ engagement in aggressive behavior to obtain attention [67], hence leading to a subsequent increase in externalizing problems.

The results on maternal overprotection also confirm previous findings showing that maternal overprotective parenting is related to greater externalizing problems [32]. Indeed, mothers’ overprotection may be perceived as intrusive parenting that may induce adolescents to reject authority and engage in oppositional and rule-breaking behavior [68]. Interestingly, unlike maternal overprotection, paternal overprotection did not predict externalizing problems. These findings warrant further research attention. Social expectations associated with maternal and paternal roles might affect how parental behaviors of overprotection are perceived and, hence, how they affect offspring development [31]. 

On the other hand, our study confirmed that parenting practices significantly predicted difficulties in emotion regulation [47,48,49,50,51], except for the dimension related to the promotion of autonomy. Overall, the role of the autonomy dimension in the development of psychopathology appears to be poor. Indeed, the promotion of the autonomy dimension did not predict either externalizing or internalizing difficulties in this study. These results are in contrast with some previous findings showing that low autonomy support predicts negative psychological outcomes—e.g., [31,34,35]. The limited contribution of the autonomy dimension to the development of psychopathology may be due to the measurement instrument. Although some studies on the psychometric properties of the PBI suggested that autonomy promotion and overprotection are two different factors [56], Parker et al. [23] identified overprotection and autonomy promotion as two opposite poles of a single factor.

This study also extends knowledge from previous research by suggesting additional mechanisms that may play a significant role in the relationship between parenting and internalizing/externalizing problems [19,20,21,22,69]. Specifically, and consistent with our hypotheses, the results indicate that the relationship between high overprotection and low maternal care with internalizing problems is fully mediated by difficulties in emotion regulation. Furthermore, emotional dysregulation partially mediated the relationship between paternal care and internalizing problems. These results are consistent with previous studies showing that parental care and affection significantly predict lower levels of internalizing problems and that this effect is explained by adolescents’ emotional regulation in the general population [54] and clinical [52] samples of adolescents. 

Our results also suggest that emotion dysregulation is the mechanism through which low parental care and high maternal overprotection increase the levels of externalizing problems among adolescents. These results are in line with findings reported by Walton et al. [53] but in contrast with those by Boullion et al. [54], who found that parental warmth did not predict externalizing disorders. Important methodological differences across the studies (e.g., in the research design and assessment tools) could explain these divergences which, hence, deserve further attention in future studies. 

Overall, according to the above findings and in line with the literature, it is possible to hypothesize that adolescents who perceive their parents as warm and less intrusive (overprotective) may feel easier to disclosure their feelings and emotions using emotional regulation strategies that allow them to positively cope with stressors [70], thus reducing the risk for internalizing and externalizing problems. In particular, these parenting styles seem to promote adaptive ways of relating to unpleasant emotions, including acceptance responses, the ability to discriminate and identify different emotions, and the ability to control behavior and pursue meaningful activities even when coping with unpleasant internal events [59].

Thus, preventive programs aimed at increasing parental care and promoting an adequate age-related level of protection may lead to the development of adaptive ways of responding to emotions [47,48,49,50,51], which in turn may reduce the risk of youth maladjustment [71]. For instance, the CONNECT parent group program [72] could be useful to help parents to face common challenges in the parent–adolescent relationship. Previous studies showed that this program is effective in reducing adolescent internalizing and externalizing problems up to the 4-month follow-up [73]. 

Moreover, there are promising psychological interventions aimed at promoting adaptive emotional regulation strategies in both parents and adolescents, such as Acceptance and Commitment Therapy (ACT) [74,75]. ACT-based interventions focus on helping people develop useful abilities to relate to unpleasant thoughts and feelings, such as acceptance, cognitive fusion, or flexible present-focused attention. 

Although interesting, this study presents some limitations. Firstly, the sample size was small and consisted of voluntary students; future research with larger and both community and clinical samples is needed to enhance the generalizability of our findings. 

Second, we relied solely on self-reported emotional regulation skills, parenting, and behavioral problems. While widely used, self-report measures are subject to limitations such as social desirability and lack of awareness, which can underestimate what actual responses are. Future research on this topic could include measures of these confounds and explore mediational mechanisms using a multimethod assessment approach (i.e., measuring variables through different informants, such as parents and/or teachers, in a variety of contexts, and using a variety of tools, including interviews and observations).

Third, future studies could also address whether the indirect effect of parenting on adolescents’ mental health remains when controlling for potential confounders such as parental distress or high adolescent negative emotionality, which have been shown to significantly predict dysfunctional parenting styles and negative psychological outcomes in childhood and adolescence [76,77].

Finally, this is a cross-sectional study and, therefore, our results are not sufficient to support temporal relationships among the variables. Prospective and longitudinal studies are needed to further examine the mediational role of emotion dysregulation in the relationship between parenting and emotional/behavioral problems in adolescents. Research on mediation is particularly important in testing theoretical models since mediation constitutes the basis of most theories. Furthermore, the findings from mediation studies may be useful to enhance the effectiveness of interventions aimed at preventing and treating psychological disorders since interventions are designed to change the outcome of interest by targeting mediating variables. 

## Figures and Tables

**Table 1:** Pearson’s correlation coefficients for the main variables in this study (N = 104).

Variables	1	2	3	4	5	6	7	8	9	10	11
1. Gender ^+^	-										
2. Age	0.07	-									
3. Paternal care	0.00	−0.02	-								
4. Paternal promotion of autonomy	0.08	0.18	0.36 ***	-							
5. Paternal overprotection	0.05	−0.05	−0.39 ***	−0.58 ***	-						
6. Maternal care	−0.08	0.04	0.39 ***	0.15	−0.07	-					
7. Maternal promotion of autonomy	0.08	0.35 ***	0.26 **	0.57 ***	−0.19	0.29 **	-				
8. Maternal overprotection	−0.13	−0.11	−0.32 ***	−0.30 **	0.35 ***	−0.40 ***	−0.46 ***	-			
9. Difficulties in emotion regulation	0.04	−0.16	−0.40 ***	−0.19	0.30 **	−0.32 ***	−0.22 *	0.27 **	-		
10. Internalizing problems	0.27 **	−0.06	−0.40 ***	−0.17	0.28 **	−0.28 **	−0.10	0.16	0.66 ***	-	
11. Externalizing problems	−0.28 **	−0.22 *	−0.25 **	−0.15	0.15	−0.21 *	−0.24 **	0.24 *	0.39 ***	0.24 *	-
Mean	-	15.62	21.76	13.10	2.95	25.25	12.46	3.27	50.62	22.10	13.66
SD	-	1.38	8.13	3.86	2.60	5.94	3.40	2.53	14.66	10.31	7.45
Range	-	14–18	0–33	0–18	0–11	9–33	1–18	0–12	26–92	3–50	1–37

* *p* ≤ 0.05; ** *p* ≤ 0.01; *** *p* ≤ 0.001. ^+^ Dummy variable: 0 = male; 1 = female.

**Table 2:** Regression analyses to explore whether parenting dimensions predict internalizing and externalizing problems ^+^.

Predictor	Dependent Variable	*β*	t	Adjusted R^2^	*F*	△R^2^	*F* Change
Maternal care	Internalizing	−0.25	−2.68 **	0.13	5.84 ***	0.06	7.19 **
Maternal autonomy	Internalizing	−0.10	−0.94	0.07	3.54 *	0.01	0.88
Maternal overprotection	Internalizing	0.19	2.01 *	0.10	9.32 **	0.04	4.03 *
Paternal care	Internalizing	−0.40	−4.45 ***	0.20	9.32 ***	0.16	19.79 ***
Paternal autonomy	Internalizing	−0.19	−1.89	0.07	3.54 *	0.03	3.57
Paternal overprotection	Internalizing	0.26	2.78 **	0.11	5.02 **	0.07	7.73 **
Maternal care	Externalizing	−0.22	−2.40 *	0.15	6.73 ***	0.05	5.78 *
Maternal autonomy	Externalizing	−0.16	−1.65	0.12	5.56 ***	0.02	2.71
Maternal overprotection	Externalizing	0.19	1.97 *	0.13	6.00 ***	0.03	3.86 *
Paternal care	Externalizing	−0.26	−2.82 **	0.17	7.73 ***	0.07	7.98 **
Paternal autonomy	Externalizing	−0.10	−1.01	0.11	5.07 **	0.01	1.01
Paternal overprotection	Externalizing	0.15	1.62	0.12	5.69 ***	0.02	2.61

^+^ *F* for the final model; *β* and t values for the last step. * *p* ≤ 0.05; ** *p* ≤ 0.01; *** *p* ≤ 0.001.

**Table 3:** Regression analyses to explore whether parenting dimensions predict difficulties in emotion regulation ^+^.

Predictor	*β*	t	Adjusted R^2^	*F*	△R^2^	*F* Change
Maternal care	−0.31	−3.27 ***	0.10	4.71 **	0.10	10.69 ***
Maternal promotion of autonomy	−0.19	−1.83	0.03	2.19	0.03	3.37
Maternal overprotection	0.26	2.69 **	0.07	3.51 *	0.07	7.21 **
Paternal care	−0.40	−4.41 ***	0.16	7.46 ***	0.16	19.42 ***
Paternal promotion of autonomy	−0.17	−1.70	0.02	1.81	0.03	2.88
Paternal overprotection	0.29	3.03 **	0.08	3.96 **	0.08	9.19 **

^+^ *F* for the final model; *β* and t values for the last step. * *p* ≤ 0.05; **p ≤ 0.01; ***p ≤ 0.001.

**Table 4:** Regression analyses examining the last step for testing emotion regulation as a mediator in the relationship between parenting styles and internalizing/externalizing problems ^+^.

Predictor	Dependent Variable	*β*	*t*	Adjusted R^2^	*F*	△R^2^	*F* Change
Maternal care	Internalizing	−0.05	−0.71	0.48	24.55 ***	0.41	40.36 ***
Difficulties in emotion regulation		0.63	8.28 ***				
Maternal overprotection	Internalizing	0.02	0.32	0.48	24.34 ***	0.41	39.98 ***
Difficulties in emotion regulation		0.65	8.54 ***				
Paternal care	Internalizing	−0.16	−2.05 *	0.48	24.31 ***	0.44	42.33 ***
Difficulties in emotion regulation		0.59	7.36 ***				
Paternal overprotection	Internalizing	0.08	1.05	0.46	22.82 ***	0.42	39.53 ***
Difficulties in emotion regulation		0.63	8.14 ***				
Maternal care Difficulties in emotion regulation	Externalizing	−0.12 0.34	−1.27 3.68 ***	0.24	9.08 ***	0.15	10.04 ***
Maternal overprotection	Externalizing	0.09	1.02	0.24	8.89 ***	0.15	9.69 ***
Difficulties in emotion regulation		0.35	3.87 ***				
Paternal care	Externalizing	−0.13	−1.36	0.24	9.14 ***	0.15	9.89 ***
Difficulties in emotion regulation		0.32	3.32 ***				

^+^ *F* for the final model; *β* and t values for the last step. * *p* ≤ 0.05; *** *p* ≤ 0.001.

## Data Availability

The data presented in this study are available on request from the corresponding author. The data are not publicly available due to privacy.
